# 

**Published:** 1887-11-12

**Authors:** 


### Notice to Advertisers.

The scale of charges for small prepaid Advertisements—Situations Wanted, Situations Vacant, &c , &c., is as follows :—

| | s. | d. |
|---|---|---|
| One insertion of three lines | 1 | 0 |
| Three „ „ „ | 2 | 6 |
| Per line afterwards | 0 | 6 |

*A line averages ten words.*

All copy for Advertisements must be sent to A. P. Watt, 2, Paternoster Square, E.C., by first post on the Tuesday *preceding* publication.

### NOTICE TO CORRESPONDENTS.

All correspondence must be written on one side of the paper only. We cannot undertake to return MSS. not used.

**Communications**, whether intended for insertion or for private information, must be authenticated by the names and addresses of the writers, not necessarily for publication.

**Local papers** containing reports or news paragraphs **should be marked.**

It is especially requested that early intelligence of **local events of interest**, or which it may be thought desirable to bring under notice, may be sent direct to the Editor.

Communications respecting editorial matters should be addressed to the Editor, Norfolk House, Norfolk Street, Strand, London, W.C.

### AMUSEMENTS WITH PROFIT FOR ALL AGES.

**Rules.**—All answers must be addressed to the PRIZE EDITOR, THE HOSPITAL, Norfolk House, Norfolk Street, Strand, W.C., not later than the *first* post on Friday next. The full name and address must in all cases be given, but a *nom de plume* may be added for publication. Only one side of the paper must be written on.

No one will be permitted to win the Monthly or Quarterly Prizes twice in any one year, the annual prizes being offered especially to prevent this.

### FOR MEN AND WOMEN.

**I.—The Quilter Prizes. Six Guineas.**

Mr. Cuthbert Quilter, M.P., has placed certain funds at our disposal, as already announced, some of which we shall expend upon prizes in this column. Prizes will be given

**For best Specimens of Work.**

Three prizes of three guineas, two guineas, and one guinea, will be given to the competitor who sends in the first, second, and third best specimens of work of any kind, suitable for presentation to the inmates of the Hospitals, addressed to the Editor, The Lodge, Porchester Square, W., on or before the 31st of December next. This work may take the form of a toy, a doll, or a scrap book for the children; an article of clothing, a painting or watercolour, an illuminated card or any other gift, for men or women, or for to aid decoration. It is proposed to arrange for the distribution of the articles sent in for this competition amongst the patients in the hospitals.

**II.—Word and Literary Competition.**

Commenced November 5th, 1887; ends January 28th, 1888.

Three prizes of one guinea, 15*s.* and 10*s.* 6*d.* will be given each quarter to the three competitors who obtain the highest number of marks: (1) by making the largest number of words derived from the word or phrase set for anatomical dissection; or (2) for the best answers to (6); or (3) by (1) and (2) combined.

(A) THE WORD COMPETITION.

We shall give the newspapers for disection during the first quarter, that for this week being

"THE ILLUSTRATED LONDON NEWS."

OBSERVE.—Proper names, abbreviations, foreign words, words of less than four letters and repetitions are barred. Nuttall's dictionary only to be used.

(B) THE LITERARY COMPETITION.

Some people hate puzzles of all kinds, including word competitions, but they are fond of correspondence, and many sisters and mothers are continually receiving and sending letters to and from members of the family who are abroad in India or the Colonies. The constant coming and going between England and other portions of the British Empire make it a matter of interest and importance to know something about the life, the climate, the amusements, the adventures, the clothing, and the surroundings of those who seek a livelihood beyond the seas. We shall therefore try to find space for the publication of selected letters from abroad, which may be sent by any of our readers who have friends and relations in foreign lands. We shall give marks for these letters as well as for the word competitions which will count equally for these prizes, so that anyone may enter for both or either, the prizes to be given to those who get the highest number of marks. The first letter from across the seas will be found on page 93 of THE HOSPITAL for Nov. 5th last. It is a record of adventure in Australia, and gives a good idea of one class of letter suitable for this column.

**Entertainments at Hospitals and Asylums.**

We propose to give prizes of one guinea and of half-a-guinea respectively to the correspondents who send from time to time between now and the 31st of March next the best, brightest, and briefest descriptions of concerts, theatrical and other entertainments given during the winter to the inmates of any public institution.

NOTE.—No coupons need be sent in connection with any of these competitions, and we hope they are sufficiently varied to induce a large number of persons to enter for them. The Quilter Prizes No. I. will be open until 31st December, 1887. Competition No. II. will end on January 28th, 1888.

**Results of Third Quarterly Competition.**

As MATER and CONDORCET have agreed to divide the prize, we award MATER (Mrs. Reeve, Barton-under-Needwood) 1½ guinea; CONDORCET (Miss C. Montgomery, Sherwood House, 26, Pembroke Gardens, W.); to whom the prizes have been forwarded.

### THE THEATRES.

SEVERAL pieces have run their time and have been withdrawn, and amongst the most notable of these are *Sophia*, which has been replaced at the Vaudeville by *Heart of Hearts*; *Ruddigore*, which is to be followed this evening by a revival of *Pinafore* at the Savoy, and *The Doctor*, which was replaced last Saturday evening by *The Arabian Nights*. Other changes are announced which will mean the taking off of several popular pieces for the purpose of making room for Christmas novelties. Of these pieces *Esmeralda*, at the Gaiety, which will run for only five more weeks, ought certainly to be seen by those who enjoy good songs, lively choruses, pretty stage effects, and a piece with plenty of go in it. Like all other burlesques, *Esmeralda* has little plot; indeed, it may be said no plot at all. Mr. Lonnen takes the principal part in the play, and makes up in a quaint way as a scheming monk, seeking to win the hand of the charming Esmeralda (Miss Marion Hood) from her faithful young lover, Phœbus (Miss Fannie Leslie). Miss Leslie's performance is disappointing, and certainly falls below some of the representations Miss Nellie Farren, whose absence leaves a marked blank, has given. Miss Hood looks charming, and does well in the light part she has, but there is not much of it. "Killaloe," as sung by Lonnen, is the hit in the piece, and fairly brings down the house. If possible, it is even more popular than his famous song "Ballyhooly," in *Monte Cristo*. The choruses are good and the dancing fair.

At the Comedy Theatre, in Panton Street, *The Barrister* is drawing large houses every evening, and creates much laughter. It is being advertised by all sorts of ingenious means, such as men thrusting large blue papers, surprisingly like writs, into the hands of unsuspecting passers by, and by men in the full dress of barristers driving round the town. It is of the *Private Secretary* order of piece, and the authors have laid themselves out to make a joke honestly if they can, if they can't do so honestly yet must they do it. Those who like the sort of piece may go and watch the troubles of a man who has unwittingly taken another's rooms as let to him by a dishonest servant, has a midnight adventure with a lady, in the course of which he exchanges his bag for hers, and so the troubles begin; for when he searches for his law papers with the idea of mastering a difficult case he finds only a pair of stays—*vide* advertisements everywhere. The acting is very equal all through the piece, but there is nobody who has any particular opportunity of making a mark.

The Avenue Theatre is open again with a new opera, adapted by Farnie, music by Planquette. This theatre has remained faithful to opera bouffe distinct from the Gilbert-Sullivan order, and as usual depends very much upon Mr. Arthur Roberts, who is better in the *Old Guard* as Polydore Poupart (Maire of Vaudrez-les-Vignes and landlord of the Big Sabot Inn) than in any other piece he has appeared in lately. His trial scene is a distinctly clever and entertaining piece of acting, and so too is his appearance in disguise as a soldier of the Old Guard. His singing has certainly improved, and his fictitious French song is of its kind the best thing which he has ever done. Few actors can bring down the house as he can by some simple by-play or grimace. Miss Broughton acts with great spirit, and Mr. Dallas does fairly well as the Marquis D'Artemare, but Miss Marion Edgcumbe, who plays the part of Fraisette (maid of the village inn), the real daughter of the marquis, good voice as she possesses, is much too heavy in many ways, and cannot be compared to such an actress as Miss St. John in her part. Mr. Alec. Marsh is well known in the concert-room, and that he should risk spoiling his excellent baritone voice by singing falsetto notes is a great pity. Other characters are well filled, and Miss Henriette Polak makes her mark as Patatout, a charming little bugler of the Imperial Guard. The scene is France, the time Napoleon's reign, and the cause of troubles his desire to marry his upstart commanders into the old families of France, but all ends happily.

### THE CHILDREN'S CORNER.

**Second Monthly Competition.**

PUZZLES.—SECOND WEEK.

No. 5. A DINNER ENIGMATICALLY EXPRESSED.

SOUPS.
A box and a fruit.
A science, a pronoun, to suffocate.
FISH.
A winter's amusement.
A warlike weapon.
ENTREES.
Sprees and panics.
POULTRY.
A tailor's requisite (roast).
SAUCE.
An autumn fruit.
SWEETS.
Something not worth a thought.
A colour, a pledge, acid.

No. 6. CONUNDRUM. Why is bread-sauce like a plough?

No. 7. ARITHMETICAL PUZZLE. "How old is that house," asked a boy of his father, pointing to an old-fashioned country house as they were passing. The father replied, "The square of its age 60 years ago is double its present age." How old was it?

No. 8. HISTORICAL ACROSTIC. 1. A battle in which Charles II. was routed. 2. A siege for which Richard is renowned. 3. A famous naval engagement. 4. One of Henry of Winchester's battles. 5. Wars well known in England. 6. "The battle of Nations." 7. A battle in which Henry Hotspur was made prisoner. 8. One of Marlboro's battles. The *initials* give the name of a celebrated battle.

LETTERS. Next week tell me all you can about *Holyrood Castle*.

**Results of Fifth Week Puzzles.**

No. 16. MISSING LETTER PUZZLE.

The way was long, the wind was cold,
The minstrel was infirm and old;
His wither'd cheek and tresses gray
Seemed to have known a better day.

No. 17. DIAMOND PUZZLE.

```
    D
  E R A
D R A K E
  E K E
    E
```

No. 18. FRENCH PUZZLE. Hélène est née au pays grec. Several correspondents have omitted the last word; I fear they forgot the French for **y**.

No. 19. RIDDLE-MA-REE. Cromwell.

*All right*—A. C. K., Toby, G. B. P., Tim, Scrooge. *Three right*—Fern, Paddy, Pepita, Gem, Jerusalem. *Two right.* Spider, Princess Ida, The Eda, Alsie, Poodle, A. G. S., McCulloch, Judy.

LAMBETH PALACE.

I am very much pleased with the letters on *Lambeth Palace*. The subject is not an easy one, and the best letters are from those who have taken the trouble to collect enough facts to make their letters both interesting and instructive. To those children who say, "We know nothing about these old buildings, and so cannot write a letter upon them," I would say it is for this very reason that I want you to *find out* something to tell me. If a prize is worth *gaining* it is worth *striving* for. Most of the accounts are apparently taken from some book of reference. We have no room this week.

Three marks each:—The Eda, Gem, Fern Two marks each, Princess Ida.

**Notices to Correspondents.**

*Skye and Ossian.*—Your letters and puzzles received too late to be credited.

**Results of First Monthly Competition.**

A. C. K. (Master Arthur Clifton Kelly, Ellesmere, Gratwicke Road, Worthing, aged eleven,) and G. B. P. (Master G. B. Pakenham, 4, The Mall, Armagh, Ireland, aged, 11½) having gained an equal number of marks, the prize of one pound is divided between them. We hope as the same competitors are not again eligible for the *Monthly Prize*, that they will return with renewed zest to the contest in anticipation of the yearly one of *Three Guineas*, of which they have yet a chance.

### Hospitals, Doctors, Treatment, and Attendance.

### THE IN- AND OUT-PATIENTS' HOSPITAL DIARY.

This Diary is so arranged as to make some portion of it appear every week, and the whole in each monthly part. It has been very difficult to compile, and corrections will at all times be gratefully received. It has been suggested that similar information about the larger Provincial and Scotch Hospitals would be useful to many readers. We should be glad to hear if this is felt to be the case.

### I.—GENERAL HOSPITALS.

| Hospitals and Terms of Admission. | Physicians, Surgeons, and Medical Officers, with Days and Hours of Attendance, |
|---|---|
| **Charing Cross Hospital**, West Strand, W.C. Sec., Mr. A. E. Reade<br>Urgent cases are immediately admitted as in-patients. Subscriber's letter expected in other cases<br>Out-patients admitted without subscriber's letter first time; for continued attendance they must be obtained from the Secretary | *In-Physicians*, Drs. Pollock, M. Th.; Green, W. S.; Bruce, Tu. F. Hour, 1.30<br>*In-Surgeons*, Messrs. Barwell, M. Th.; Bellamy, Tu. F.; Bloxam, W. S. Hour, 2<br>*Out-Physicians*, Drs. Wilcocks, M.Th.; Abercrombie, Tu. F.; Lubbock, W.S.; Murray, W.S. Hour, 1.30<br>*Out-Surgeons*, Messrs Cantlie, M.Th.; J.H. Morgan, Tu. F.; Stanley Boyd, W. S. Hour, 1.30 |
| **"Dreadnought" Hospital** (Seamen's Hospital Society), Greenwich, S.E. Sec., Mr. P. J. Michelli<br>Entirely free to seamen and to all accident cases | *In- and Out-Physicians*, Drs. Curnow, Tu. Th. S.; H. T. Griffiths, M. W. F. Daily, between 9 and 3<br>*In- and Out-Surgeons*, Messrs. W. J. Smith, G. R. Turner. Daily, between 9 and 3 |
| **French Hospital**, 10, Leicester, Place Leicester Sq. W.C. Sec., Mr. E. Rimmel<br>Free to urgent cases and to all foreigners speaking French | *In-Physician*, Dr. Vintras, Tu. S. 10<br>*In-Surgeons*, Sir W. MacCormac, Tu. 9; Mr. J. Késer, W. F. 10<br>*Out-Physician*, Dr. Vintras, Tu. S. 10<br>*Out-Surgeons*, Messrs. H. de Méric, M. Th. 10; J. Késer, W. F. 10 |
| **German Hospital**, 113, Dalston Lane, E. Sec., Mr. C. Feldmann<br>Free to natives of Germany and others speaking German, and to all accidents and emergencies. Others by Governors' letters.<br>———Eastern Branch, 49, Mansell Strset, Aldgate, E.<br>———Western Branch, 259, Oxford Street, W. | *In-Physicians*, Drs. Weber, M. T. 2; Port, Tu. F. 9 a.m.<br>*In-Surgeons*, Drs. Lichtenberg and Burger, W. S. 2<br>*Out-Physicians*, Drs. Tuchmann, Tu. W. 2; Ludwig, M. Th. 2; Keller, Tu. W. F. 2; Phillippi, M. Th. F. 2 (*Men*, Tu. Th.; *Women*, M. W. F.)<br>*Out Physician*, Dr. C. Harrer, Tu. F. 8 to 10 a.m.<br>*Out-Physician*, Dr. M. Castaneda, M. T. 8 to 10 a.m. |
| **Great Northern Central Hospital**, Caledonian Rd., N. Sec, Mr. W. T. Grant<br>Free to all, without letters of recommendation | *In-Physicians*, Drs. Cholmeley, Cook, and Burnett, M. Tu. W. Th. F. 2<br>*In-Surgeons*, Messrs. W. Adams, W. Spencer Watson, and J. Macready, M. Tu. W. Th. F. 2<br>*Out-Physicians*, Drs. Beale, M. Th. 2; Beevor, Tu. 2, S. 10<br>*Out-Surgeons*, Messrs. Lockwood, M. Th. 2; Makins, Tu. F. 2 |
| **Guy's Hospital**, S. Thomas's Street, S.E. Sup., Dr. C. J. Steele<br>Patients are admitted, if beds are available, without letters of recommendation, on application at the Hospital. Out-patients are seen free without letters of recommendation | *In Physicians*, Drs. Pavy, Tu. F.; Pye-Smith, M. Th. S.; Taylor, M. Tu. Th. F. Hour, 1.30<br>*In-Surgeons*, Messrs. Bryant, M. Th. Durham, M. Th. F.; Howse, W. S.; D. Colley, M. Th. Hour, 1.30<br>*Out-Physicians*, Drs. Carrington, W.; Hale White, M.; Goodhart, M. Tu. Th. F. Hour, 12.30<br>*Out-Surgeons*, Messrs. Lucas, Th.; Golding Bird, S.; Jacobson, W.; Symonds, M. Hour, 12.30 |
| **King's College Hospital**, Portugal Street, W.C. Sec., Mr. T. Mosse Macdonald<br>Accidents and urgent cases free at all times. Other cases by Governors' letters, but where not obtainable, application may be made to the Secretary | *In-Physicians*, Drs. Beale, Duffin, Yeo, Tu. W. F. 1.30; Tu. W. F. S. 2<br>*In-Surgeons*, Mr. Wood, Sir J. Lister, Messrs. H. Smith, W. Rose. Daily, 1.30<br>*Out-Physicians*, Drs. Ferrier, J. Curnow, N. I. C. Tirard. Daily, 1<br>*Out-Surgeons*. Messrs. W. Rose, W. Cheyne, A. B. Barrow. Daily, 1 |
| **London Hospital**, Whitechapel Road, E. Sec., Mr. A. H. Haggard<br>Accidents and urgent cases free; out-patients for dental and maternity departments need no recommendation; other cases by subscriber's order. Out-patients are required to attend only once, unlesss otherwise directed by the Physician or Surgeon | *In-Physicians*, Drs. Mackenzie, Tu. F.; Down, Tu.F.; H. Jackson, M.Th; Sutton, M. Th.; Fenwick, Tu. F. 1.30<br>*In-Surgeons*, Messrs. Rivington, Tu F.; Conper, Waren Tay, M. Th.; McCarthy, W. S.; Treves, Tu. F. Hour, 1.30<br>*Out-Physicians*, Drs. Sansom, Th.; M. Ralfe, M. Th.; Turner, Tu. F; Warner, Tu. F.; Gilbart Smith, W. S.; J. Anderson, W. S. Hour, 1.30<br>*Out-Surgeons*, Messrs. Mansell-Moullin, M. Th.; Eve, M. W.; Reeves, Tu.S; H. Fenwick, Tu. F. Hour, 1.30 |
| **London Temperance Hospital**, Hampstead Rd.<br>For treatment without alcohol. By letter or small payment | *In- and Out-Physcians*, Drs. J. Edmunds, M.; J. J. Ridge, Tu. F. Hour, 1.30<br>*In- and Out-Surgeons*, Mr. A. P. Gould, Th. 2.30 |
| **Metropolitan Free Hospital**, Kingsland Road, N. Sec., Mr.<br>Admission free to the sick poor, without any letter of recommendation. Urgent cases at all times | *In- and Out-Physicians*, Drs. Drysdale, Tu. F. 12; J. G. Dudley, W. S. 12; Fotherby, M. Th. 12; H. H. Tooth, Tu. F. 9; A. Haig, W. S. 9; A. Davies, M Th. 9<br>*In- and Out-Surgeons*, Messrs. E. J. Chance, W. S. 9; Goodsall, Tu. F. 9; Walsham, M. Th. 9; A. A. Bowlby, F. 9; S. Paget, Th. 9; C. J. Thompson, daily, 9 |

| Hospitals and Terms of Admission. | Physicians, Surgeons, and Medical Officers, with Days and Hours of Attendance. |
|---|---|
| **Middlesex Hospital**, Berners Street, W. Sec. and Supt., Mr. A. O. D. Bartholeyns<br>Urgent cases admitted at all times; other cases by ticket. All medical cases must, in the first instance, be seen by the Resident Medical Officer, who attends at the hospital daily, at 11 a.m. | *In-Physicians*, Drs. Cayley, M. Th.; Coupland, Tu. F.; D. Powell, M. Th.; Finlay, Tu. F. Hour 1.30<br>*In-Surgeons*, Messrs. Hulke, M. Th.; Lawson M. Th.; Morris, Tu. F. Hour, 1.30<br>*Out-Physicians*, Drs. Fowler, Tu. F. 3.30; Biss. M. Th. 1.30; Pringle, W. S. 1.30<br>*Out-Surgeons*, Messrs. Andrew Clark, M. 1.15 (*Men*); Th. 1.15 (*Women*); Pearce Gould, F. 1.30 (*Men*); Tu. 1.30 (*Women*); J. B. Sutton, W. S. 9 |
| **Miller Hospital and Kent Dispensary**, Greenwich, S.E. Hon Sec., Mr. W. Bristow. | *Physicians*, Drs. T. Creed, C. H. Hartt, G. Wiles<br>*Surgeons*, Messrs. T. Moore, J. Poland, E. Clarke |
| **North-West London Hospital**, 18, Kentish Town Road, N.W. Sec., Mr. A. Craske<br>By subscribers' tickets or payment, but accidents and urgent cases free at all times | *In- and Out-Physicians*, Drs. W. C. Hood, J. Shaw, Edwarde H. Campbell. Daily 1.30<br>*In- and Out-Surgeons*, Messrs. F. Durham Collier, Jennings, J. Black. Daily 1.30 |
| **Ospedale Italiano**, 41, Queen Square, Bloomsbury. Sec., Sig. L. Bonacina<br>Free to all, but Italians have preference | *In- and Out-Physicians*, Drs. M. Castaneda, Tu F. 10 to 11; B. Sassella, M. Th 10 to 11; Mr. J. W. B. Steggall, W. S. 4 to 5 |
| **Poplar Hospital**, 303 East India Dock Road, E. Sec., Lt-Col. Feneran<br>Free to accidents and emergencies | *In-Surgeons*, Messrs. F. M. Corner, M. Brownfield and Resident Surgeons<br>*Out-Surgeons*, Dr. J. D. Grant, W. S.; Mr. T. E. Bowkett, Tu. F. 12 to 2 |
| **Royal Free Hospital**, Gray's Inn Road, W.C. Sec., Mr. J. S. Blyth<br>Admission free to the sick poor without any letter of recommendation | *In- and Out-Physicians*, Drs. Cockle, M. Th.; Sainsbury, Tu. F.; West, W. S. Hour, 1<br>*In- and Out-Surgeons*, Messrs. Rose, M. Th.; W. Gant, Tu. F.; Barrow, W. S. Hour, 1 |
| **St. Bartholomew's Hospital**, Smithfield. Clerk, Mr. W. Cross<br>Admission free, without letter or ticket. Accidents and other urgent cases admitted at any time; ordinary cases of disease daily, between 9 and 10. Patients are seen for Electrical treatment on M. Tu. Th. and F. 1.30 | *In-Physicians*, Drs. Andrew, Church, Gee, Sir D Duckworth. Daily, 1.30<br>*In-Surgeons*, Messrs. Savory, T. Smith, Willett, Langton, M. Baker. Daily, 1.30<br>*Out-Physicians*, Drs. Hensley, M. Th. 11; Brunton, Tu. F. 11; Legg, W. S. 11; N. Moore, Tu. W. Th. S. 9<br>*Out Surgeons*, Messrs. Marsh, Tu. F. 12 Butlin, M. Th. 12; Walsham, W. S. 12 Cripps and Bruce Clarke, daily, 9<br>*Casualty Physicians*, Drs. Davies, Tu. W. F. S.; O. Browne, M. Tu. Th. F.; Nias, M. W. Th. S. Hour, 9 |
| **St. George's Hospital**, Hyde Park Corner, S.W. Sec., Mr. C. L. Todd<br>Free to accidents and emergencies. Other in-cases by Governor's or subscriber's letter, but, in necessitous cases, this recommendation is not strictly insisted on. Letters are not required for out-patients. Vaccination Th. at 11 | *In-Physicians*, Drs. Wadham, M. F.; Dickinson, M. Tu. Th. F.; Whipham, Tu. W. S.; Cavafy, Tu. S. Hour, 1<br>*In-Surgeons*, Messrs. Holmes, M. Th. F. 1, W. 1.30; Rouse, M. Th. F. 1, W. 1.30; Haward, Tu. Th. S. 1. W. 1.30<br>*Out-Physicians*, Drs. Ewart, M. F.; Owen, Tu S. Hours, 10.30 to 12<br>*Out-Surgeons* Messrs. Bennett, M. F.; Dent Tu. S. Hours 10.30 to 12<br>*Men*, F. S.; *Women*, M. Tu. |
| **St. Mary's Hospital**, Cambridge Place, Paddington, W. Sec., Mr. Thomas Ryan.<br>In-patients are admitted by letter of recommendation. Urgent cases and accidents free at all times. Out-patients require a letter. Electrical treatment Tu. and F. 2 | *In-Physicians*, Sir Edward Sieveking, Tu. F. 1.45; Drs. Broadbent, M. Th. 1.45; Cheadle, W. 1.45, S. 9 30<br>*In-Surgeons*, Messrs. Norton, M. Th. 1.45; Owen, Tu. F. 1.45; H. Page, W. S. 1.45.<br>*Out-Physicians*, Drs. D. B. Lees, W. S.; S. Phillips, M. Th.; R. Macguire, Tu. F. Hour, 1.30<br>*Out-Surgeons*, Messrs. W. Pye, M. Th.; A. J. Pepper, Tu. F.; A. Q. Silcock, W. S. Hour, 1.30 |
| **St. Thomas's Hospital**, Westminster Bridge Road, S.E. Steward, Mr. F. Walker<br>Accidents and emergencies free at all times. Other cases by Governor's letter, but, where unobtainable, application should be made to the Steward by letter, or personally between 10 and 4 Vaccination on W. at 11.30 | *In-Physicians*, Drs. Bristowe, Tu. F.; Stone, M. Th.; Ord, M. Th.; J. Harley, Tu F.; Gervis, Tu. F. Hour, 2<br>*In-Surgeons*, Messrs. Sydney Jones, Tu. F.; Croft, M. Th.; Sir W. MacCormac, M. Th. Hour, 2<br>*Out-Physicians*, Drs. Payne, Tu. F.; Sharkey, M. Th.; Gulliver, W. S. Hour, 1.30<br>*Out-Surgeons*, Messrs. Clutton, Tu. F.; Anderson, W. S.; Pitts, M. Tu. Hour, 1.30 |
| **Tottenham Training Hospital**, Tottenham, N. Director, Dr. Laseron<br>Free. Accidents and emergencies admitted at all times | *In-Physician*, Dr. Rasch, Tu. F. 3 to 5<br>*In-Surgeons*, Drs. Lichtenberg, M. Th. 3 to 5; E. H. May, M. Th. 3 to 5<br>*Out-Surgeon*, Dr. Lloyd Smith, M.W. 11 to 2; *Men* only, W. 7 to 8 p.m. |

### THE STUDENTS' COLUMN.

### ROYAL COLLEGES OF PHYSICIANS AND SURGEONS, EDINBURGH, AND FACULTY OF PHYSICIANS AND SURGEONS, GLASGOW.

The triple qualification examinations were held in Edinburgh, in October, with the following results:—

Passed First Examination.—Roger Bernard Burke, Cork; William Henry Helm, London; Denis Michael Bernard Myers, Clonmel; Richard Francis Shaw, Hereford; Andrew George Rolland, North Shields; Robert Tucker Fallon, Westminster; John Birtwhistle Griffiths, Stroud; Thomas Henry Hayton, Cumberland; Edmund Denison, Leeds; William Arthur Collier, Donegal; John Alexander McDonald, Kanturk; Andrew Hunter Graham, Omagh; Angus Livingstone McDougal, Ross-shire; Mourchide Sheik-Hossen Camal Boudou, Mauritius; Francis Hugh Scott, Belfast; Vernon Francis Allen, County Cork; Thomas Henwood Adams, Cornwall; Robert Smyth, S. Australia; Robert McCoull, Northumberland; Thomas Ross Scott, Edinburgh; Henry David Gregory Chambers, Lee; Bernard Tomkys, Bilston; John Walter Corns, Manchester; Joseph Wilson, Newry; Michael Luby, County Limerick; Robert Day Stokes, Woolwich; Peter Wright McGregor, Glasgow; Edwin Callender, Haydon Bridge; Godfrey William Waller de Courcy Baldwin, County Tipperary: Stuart Charles Bloxham, Halesowen; Allison Albany Davies Parker, Monmouthshire; Frank Provis Dodd-Thomas, Chester; William Cornelius Rainsbury, County Cork; Charles Stewart, Midlothian; William MacKenzie, Banffshire; Henry Carr, Dublin; Lachlan Macdonald, Inverness-shire; Frederick William Marshall, Staffordshire; Simon Fraser, Inverness-shire; Augustus Lower Paliologus, Calcutta; John Dale, Dumfries; John Charles Burke, County Clare; John Heddle, Adelaide; Joseph Vincent Somers, County Tipperary; Arthur William Hogg, Leeds; Samuel Wilson, Hanley.

Passed Second Examination.—Arthur William Hogg, London; William Dwyer Russell, Kilross; Benjamin Evans Jones, Crewe; Henry Edwin Scott, Edinburgh; Thomas Henry Hibbins, Ketton; Harvey Macpherson, Cheltenham; John Ferris, Devon; Roger Bernard Burke, Cork; James Murphy, County Limerick; Charles Howard Jackman, Herefordshire; John Cowe, Roxburghshire; Miss Evelina Ada Cargill, London; William Henry Helm, London; Samuel Taite Beckitt, Liverpool; Charles Smyth, County Limerick; Robert Vincent Sutton, Cork; Francis George Heard, Cork; David Montgomery Paton, Ayrshire; Matthew Wiseman, County Cork; John Jackson, Cockermouth; Thomas McCubbin, Kirkintilloch; Herbert Osborne, Nottinghamshire; William Grey, Woodhorn; Herman Fermor Lawrence, Tasmania; Percy William Griffiths, South Wales; Robert George Spiller, Cork; Alexander Oswald Hibbert, Hanley; Patrick James Byrne, County Cork; John O'Neill, County Cork; Matthew Lovell Mackintosh, Sussex; John Columba Byrne, County Cork; Edward Blackwell Roberts, Mold; Patrick O'Donoghue, County Cork; Thomas Edward Pakenham Pollard, County Clare; Michael Gorman, Cork; William Matthews Joyce, Ashby-de-la-Zouch; Richard Francis Shaw, Hereford; William Yorke, Glasgow; Cyril Herbert Sharpe, Norwich; John Parlane Granger, Glasgow; Cuthbert Stanislaus Morrison, India; James Gemmell Curdie, Kilmarnock; James Edward Gribble, Ballarat.

Passed Third Examination and Admitted L.R.C.P., Edinburgh; L.R.C.S., Edinburgh; and L.F.P. and S., Glasgow.—Rupert George Naylor, Calcutta; John Spencer, Donegal; Miss Jane Louisa Jarrett Haskew, Calcutta; Andrew Mossforth Neatby, London; George Evan Jones, North Wales; Edward Linton, Edinburgh; Daniel Mitchell, Canada; George Dunn Wilson, County Cork; Ephraim Hilliard, Tralee; Joseph Thomas Roberts, Hanley; Arthur William Hogg, London; Joseph Stirling Greer, County Down; James David Thorburn, Canada; Thomas Harling, jun., Stockton-on-Tees; William Stewart Kidd, Alyth; Elias Clouse, Ontario; James Booth, Queensland; Richard Ambler, Hemel Hempstead; Charles Benjamin Mather, Kent; Miss Alexandrina Matilda Macphail, Skye; John Tighe, Ireland; James Thomas Moore Giffen, County Antrim; Herbert Charles Faulke, Norfolk; Daniel Henderson, County Armagh; Henry Herbert Atter, Stamford; David Hennessy Tweedie, Newry; Miss Jean Helen Grant, New York; Matthew Donoghue Gray, Ballyduffy; William Mabson Gabriel, Kendal; Abraham John Howlin, County Wexford; Charles James Lownds, Rajpootana; James Andrew Murray, Cork; James Wallace, Antrim; John McGinn, Omagh; William Henry Helm, London; Tennent Ronalds, Edinburgh; John McElfatrich, Ireland; Tom Clark, Penrith; Adam Thomson, Ontario; William Wyatt Shrubshall, Margate; Thomas Quaid Ambrose, County Limerick; Charles Leonard Ashby, Cambridge; John Phillips, Sunderland; Thomas Cussen, County Limerick; Caleb Schnehage, Orange Free State

### ROYAL COLLEGE of SURGEONS, EDINBURGH

During the October sittings of the Examiners, Walter Stanley, of Walsall, was admitted a Licentiate in Surgery, and the following gentlemen, having passed the final examination, were admitted Licentiates in Dental Surgery of the College: Ernest Edmund Taylor, Manchester; John Girdwood, Edinburgh; John Bain, Galashiels.

